# Targeting Resistance Pathways in Breast Cancer Through Precision Oncology: Nanotechnology and Immune Modulation Approaches

**DOI:** 10.3390/biomedicines13071691

**Published:** 2025-07-10

**Authors:** Hussein Sabit, Sanaa Rashwan, Yasser Albrahim, Al-Hassan Soliman Wadan, Faisal Radwan, Amany I. Alqosaibi, Shaimaa Abdel-Ghany, Borros Arneth

**Affiliations:** 1 Department of Medical Biotechnology, College of Biotechnology, Misr University for Science and Technology, P.O. Box 77, Giza 3237101, Egypt; 2 University Hospital of Leicester NHS Trust, Leicester LE5 4PW, UK; 3 Ministry of Health, Alahsa 39182, Saudi Arabia; 4 Oral Biology Department, Faculty of Dentistry, Galala University, Suez 15888, Egypt; 5 Center for Coastal Environmental Health and Biomolecular Research, NCCOS/NOS/NOAA, Charleston, SC 29412, USA; 6 Department of Biology, College of Science, Imam Abdulrahman bin Faisal University, Dammam 31441, Saudi Arabia; 7 Department of Environmental Biotechnology, College of Biotechnology, Misr University for Science and Technology, P.O. Box 77, Giza 3237101, Egypt; 8 Institute of Laboratory Medicine and Pathobiochemistry, Molecular Diagnostics, Hospital of the Universities of Giessen and Marburg (UKGM), Philipps University Marburg, Baldinger Str, 35042 Marburg, Germany; 9 Institute of Laboratory Medicine and Pathobiochemistry, Molecular Diagnostics, Hospital of the Universities of Giessen and Marburg (UKGM), Justus Liebig University Giessen, 35392 Giessen, Germany

**Keywords:** breast cancer, drug resistance, nanotechnology, immune modulation, precision oncology

## Abstract

According to the WHO, in 2022, there were 2.3 million women diagnosed with breast cancer (BC) and 670,000 deaths globally. BC remains the leading cause of cancer-related mortality, with therapeutic resistance representing a significant barrier to effective treatment, particularly in aggressive subtypes like triple-negative breast cancer (TNBC). This review article discusses emerging strategies to overcome resistance by integrating precision oncology, nanotechnology-based drug delivery, and immune modulation. Resistance mechanisms—such as metabolic reprogramming, tumor heterogeneity, immune evasion, autophagy, and the role of cancer stem cells—are critically examined. We highlight cutting-edge nanoplatforms that co-deliver chemotherapeutics and immune stimulants with spatiotemporal precision, including sonodynamic and photothermal systems, ADCs, and targeted nanoparticles. Moreover, advances in tumor microenvironment (TME) modulation, photoimmunotherapy, and exosomal miRNA targeting offer promising avenues to enhance immunogenicity and therapeutic durability. The integration of molecular profiling with advanced computational approaches, including artificial intelligence and biomimetic models, holds significant promise for the future development of personalized resistance-mitigating interventions, though a detailed exploration is beyond the current scope. Collectively, these strategies reflect a paradigm shift from conventional monotherapies toward multifaceted, precision-guided treatment approaches. This review aims to provide a comprehensive overview of current innovations and propose future directions for overcoming drug resistance in BC.

## 1. Introduction

BC remains one of the most prevalent malignancies among women worldwide and a significant cause of cancer-related mortality, with TNBC representing the most aggressive and therapeutically challenging subtype [1,2]. Despite substantial advances in early detection and treatment, the emergence of drug resistance continues to impede long-term survival across all BC subtypes [3,4]. Resistance arises through a multitude of mechanisms—including metabolic reprogramming, immune evasion, autophagy activation, and TME remodeling—that allow tumor cells to circumvent the cytotoxic effects of chemotherapy, endocrine therapy, and emerging immunotherapeutic strategies [5,6].

In recent years, precision oncology has emerged as a transformative paradigm in BC therapy, enabled by high-throughput molecular profiling and the identification of actionable genetic and epigenetic alterations (reviewed in [7,8]). Yet, translating these findings into durable therapeutic responses remains hindered by tumor heterogeneity, dynamic resistance mechanisms, and the immunosuppressive TME. Therefore, innovative strategies that integrate molecular targeting with immune modulation and nanotechnology-based delivery systems are urgently needed to overcome therapeutic resistance and achieve sustained clinical benefit.

Nanotechnology has demonstrated immense potential in revolutionizing cancer therapeutics, particularly by enabling site-specific drug delivery, minimizing systemic toxicity, and integrating multimodal therapeutic platforms. For example, ultrasound-responsive nanobubbles co-loaded with chlorin-e6 (a sonosensitizer) and paclitaxel have been shown to simultaneously mediate chemotherapy, sonodynamic therapy, and immune activation through the cGAS-STING pathway, significantly suppressing tumor progression and enhancing CD8^+^ T cell infiltration in TNBC models [9]. Similarly, single-atom iron nanozymes (Fe-N-C SAzymes) capable of mimicking peroxidase activity have been developed to remodel tumor metabolism and the immune landscape, potentiating chemodynamic therapy (CDT) and photothermal therapy (PTT) while eliminating immunosuppressive myeloid-derived suppressor cells [10].

Photothermal therapy has been further enhanced by designing nanoplatforms that incorporate immunomodulatory agents. In one such study, a novel conjugate of gold nanorods and CXCR4 antagonist peptide E5 (AuNRs-E5) not only disrupted tumor proliferation but also initiated endoplasmic reticulum stress and promoted dendritic cell maturation, resulting in long-term immune memory and suppression of TNBC recurrence [11]. Moreover, combining chemotherapy with photothermal and immune stimulation using minimalist platforms has induced robust autophagy-dependent immunogenic cell death, as evidenced by co-assembled IC/IR820 nanoparticles.

An equally compelling strategy involves the use of autologous, implantable therapeutic scaffolds has been evaluated. A recent study reported the development of a Nano–Micro-Sera-based fibrin implant incorporating photothermal agents, chemotherapeutics, and immune agonists to prevent TNBC relapse post-surgery. This multi-agent, locally administered system significantly enhanced immune infiltration at the tumor site, prevented local recurrence, and achieved remarkable tumor suppression [12].

Another prominent mechanism contributing to BC resistance is tumor cell autophagy, which can promote survival under therapeutic stress. Innovative nanoparticle designs, such as Pt(IV)/CQ/PFH constructs, have been engineered to inhibit protective autophagy while reprogramming innate immune metabolism—boosting the maturation of dendritic cells and polarization of macrophages to their pro-inflammatory phenotypes [13]. These findings underscore the therapeutic value of synchronizing metabolic reprogramming with immune modulation.

Despite the impressive progress in immunotherapy, particularly in checkpoint blockade, its clinical efficacy in BC—especially TNBC—has been hindered due to poor antigenicity and hostile immunosuppressive TME. Therefore, multi-modal nanoplatforms that integrate immunogenic cell death inducers (e.g., mitoxantrone), immune adjuvants (e.g., TLR7/8 agonists), and photothermal agents have been constructed to repolarize tumor-associated macrophages, enhance dendritic cell activation, and convert cold tumors into immunologically hot ones [14].

Collectively, these studies reflect a paradigm shift in BC therapy, moving from monotherapies to precision-guided, combination strategies that target both tumor-intrinsic resistance pathways and the extrinsic immune landscape. By leveraging nanotechnology to co-deliver chemotherapeutics, immune modulators, and photothermal agents with spatial and temporal precision, these platforms offer powerful tools to overcome tumor heterogeneity and resistance.

This review aims to comprehensively examine recent advances in nanotechnology-enabled, immune-modulatory strategies that target resistance pathways in BC, focusing on triple-negative and drug-refractory subtypes. We highlight the mechanistic basis of resistance, the role of tumor-immune crosstalk, and the clinical promise of next-generation combination therapies poised to reshape the treatment landscape.

## 2. Advances in Molecular Targeted Therapy for BC

BC remains one of the most prevalent malignancies worldwide, and despite advancements in conventional therapies, significant challenges persist in treating advanced and metastatic cases. Molecular profiling has provided insights into tumor heterogeneity, leading to the development of targeted therapies that enhance treatment efficacy and minimize resistance [15]. The introduction of precision medicine, including CDK4/6 inhibitors, immunotherapy, and antibody–drug conjugates (ADCs), has revolutionized BC management [16].

### 2.1. Molecular Profiling and Targeted Therapy in BC

**Figure 1:**
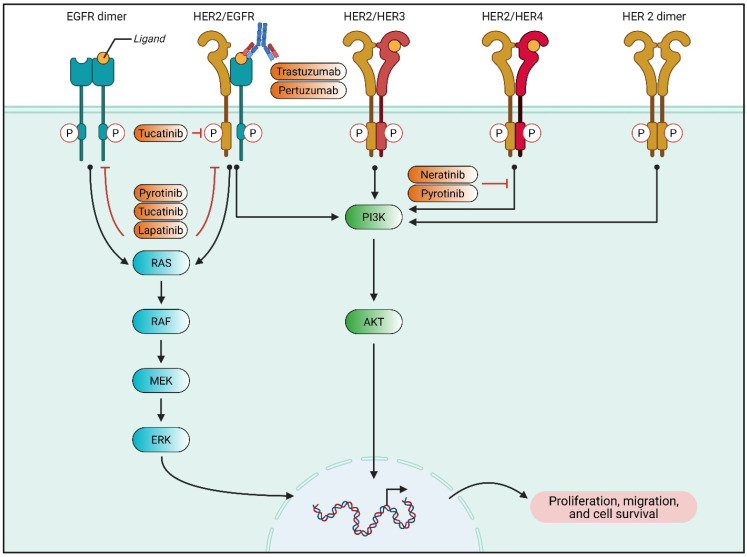
Trastuzumab and Pertuzumab suppress HER2-mediated signaling through their binding to the extracellular domain of the HER2 receptor. In contrast, small-molecule inhibitors, such as lapatinib, neratinib, tucatinib, and pyrotinib, exert their effects by targeting the intracellular tyrosine kinase domain of members of the HER receptor family. Abbreviations: EGFR—epidermal growth factor receptor; HER—human epidermal growth factor receptor; MEK—MAP kinase; ERK—MAP kinase; PI3K—phosphoinositide 3-kinases.

Molecular characterization of BC has identified key genetic alterations that drive tumor progression and therapeutic resistance. Next-generation sequencing (NGS) has facilitated the identification of oncogenic mutations, such as TP53, PIK3CA, and ESR1, which have implications for treatment selection [15]. In a study of 38 patients, molecular profiling helped the assignment of targeted therapies in 14% of cases, with a median progression-free survival (PFS) of five months. This highlights the potential of precision oncology in refining treatment regimens for hormone receptor-positive (HR+)/HER2-negative and TNBC subtypes (Figure 1).

HER2 amplification is a critical therapeutic target in BC, with HER2-targeted therapies demonstrating significant survival benefits. However, a subset of HER2-mutant but non-amplified tumors exhibit resistance to standard treatments. HER2 mutations were analyzed in 1184 patients, revealing that mutation-positive, non-amplified patients had poorer PFS prognoses than their amplification-positive counterparts. The study suggested that HER2 mutations could serve as predictive biomarkers for trastuzumab resistance, underscoring the need for alternative targeted approaches such as tyrosine kinase inhibitors like pyrotinib [17].

Similarly, the tumor suppressor gene TP53 is most frequently mutated in BC, which plays a role in therapeutic resistance. Liu, Yi [18] analyzed TP53 mutations in HER2-positive and HER2-mutant, non-amplified patients, observing that TP53 mutations correlated with shorter PFS in HER2-positive patients receiving trastuzumab. These findings suggest that TP53 mutations may be predictive biomarkers for anti-HER2 therapy response, aiding treatment stratification and developing personalized therapeutic strategies.

### 2.2. HR+/HER2− BC and the Challenge of Resistance

HR+/HER2− metastatic BC has benefited from the emergence of molecularly targeted therapies, including CDK4/6 inhibitors (palbociclib, abemaciclib) and mTOR inhibitors (everolimus). These treatments resulted in a median PFS of 5.3 months in heavily pretreated patients. Yet, resistance remains challenging, necessitating novel combination strategies and biomarker-driven interventions [19].

**Figure 2:**
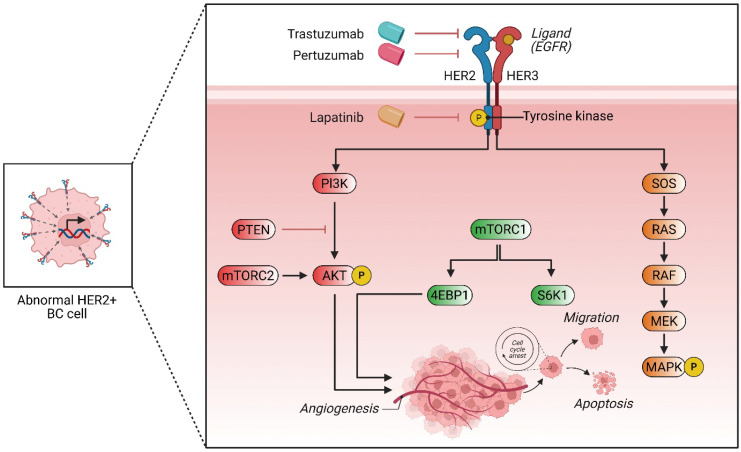
HER2+ breast cancer cell signaling pathways and therapeutic targets. This schematic illustrates the key molecular pathways activated in HER2-positive (HER2+) breast cancer (BC) cells and the mechanisms of targeted therapies. Overexpression of HER2 leads to dimerization with HER3, activating downstream signaling via its tyrosine kinase domain. Two major pathways are shown: the PI3K/AKT/mTOR pathway and the RAS/RAF/MEK/MAPK pathway. Activation of PI3K triggers AKT phosphorylation (aided by mTORC2), promoting angiogenesis and inhibiting apoptosis. PTEN acts as a tumor suppressor by inhibiting PI3K signaling. mTORC1 activates 4EBP1 and S6K1, contributing to cell growth, migration, and survival. Concurrently, HER2/HER3 activation stimulates RAS-mediated signaling through RAF, MEK, and MAPK, further supporting proliferation and survival. Targeted therapies—trastuzumab and pertuzumab—bind HER2, blocking dimerization and downstream signaling. Lapatinib, a tyrosine kinase inhibitor, inhibits phosphorylation of HER2, suppressing both PI3K and MAPK cascades. The net effect of these therapies is reduced angiogenesis, inhibited migration, increased apoptosis, and cell cycle arrest.

One of the key mechanisms of resistance in HR+/HER2− BC is the activation of alternative survival pathways, including the PI3K/AKT/mTOR axis. This has led to the development of combination therapies targeting multiple signaling cascades to delay or overcome resistance. Additionally, ESR1 mutations, commonly acquired during endocrine therapy, have been associated with reduced responsiveness to aromatase inhibitors, emphasizing the need for more effective inhibitors or alternative strategies, such as selective estrogen receptor degraders (SERDs) (Figure 2).

### 2.3. TME and Immune Modulation in Targeted Therapy

TME components, particularly tumor-infiltrating lymphocytes (TILs), influence response to targeted therapies. The ESTIMATE and CIBERSORT algorithms have been used to evaluate immune infiltration in breast tumors. The study identified a correlation between immune scores and overall survival, highlighting the potential of immune-related gene signatures in predicting trastuzumab response [20]. These findings suggest that integrating immunotherapy with molecular-targeted treatment could improve clinical outcomes.

Recent evidence suggests that tumor-associated macrophages (TAMs) contribute to drug resistance. Dong, Sun [21] demonstrated that glutathione S-transferase P1 (GSTP1)-mediated interleukin-6 (IL-6) secretion by TAMs enhances resistance in MCF-7 BC cells. Thus, targeting TAMs with immune-modulating agents may represent a viable approach to sensitize tumors to existing treatments.

### 2.4. Emerging Strategies for Drug Delivery

Recent advances in nanoparticle-based delivery systems and photothermal therapy offer promising avenues for enhancing targeted treatment efficacy. Hu, He [22] engineered mitochondria-targeted organic nanoparticles that improved cancer cell apoptosis through mild photothermal therapy, reducing tumor metastasis. Additionally, Liu, Zhou [23] showed that dual-targeted cationic microbubbles enhanced gene therapy delivery, improving tumor suppression. These novel approaches, coupled with molecular diagnostics, hold the potential for overcoming resistance mechanisms and improving treatment outcomes.

Another promising strategy involves the development of aptamer-based proteolysis-targeting chimeras (PROTACs) to combat endocrine resistance in ERα-positive BC. Feng, Zhang [24] introduced an aptamer-PROTAC approach targeting the DNA-binding domain of ERα, effectively degrading mutant ERα variants that drive resistance to tamoxifen. This novel strategy could provide a paradigm shift in the management of hormone-resistant BC.

**Figure 3:**
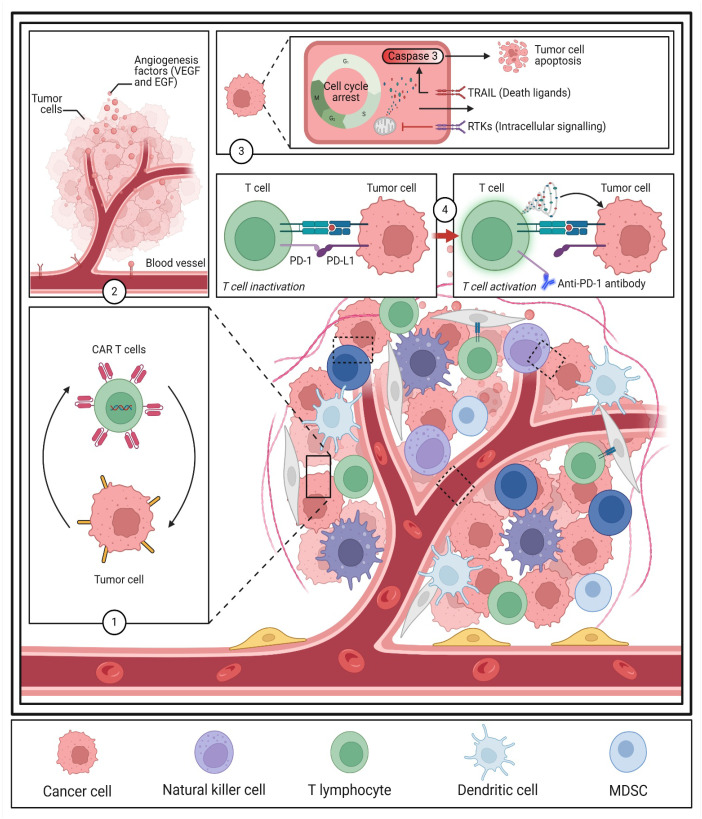
Summary of key molecular targeted therapies aimed at suppressing tumor angiogenesis, excessive growth, and immune evasion. Red labels highlight the main therapeutic targets. Abbreviations: VEGF—vascular endothelial growth factor; EGF—epidermal growth factor; TRAIL—TNF-related apoptosis-inducing ligand; RTKs—receptor tyrosine kinases; PD-1—programmed cell death protein 1; PD-L1—programmed death-ligand.

Molecular targeted therapy has transformed BC treatment, offering more precise and effective therapeutic options (Figure 3). Nonetheless, challenges such as resistance and tumor heterogeneity necessitate ongoing research into predictive biomarkers and combination strategies [25,26,27]. Integrating genomic profiling, immune modulation, and advanced drug delivery systems will be pivotal in optimizing targeted therapies and improving patient prognosis. Future research should focus on developing robust biomarker-driven approaches and refining precision medicine strategies to ensure durable treatment responses in BC patients [28,29,30].

## 3. Advances in Targeted Therapy for BC

Advances in molecular characterization and targeted therapy have significantly improved patient outcomes, particularly in HER2-positive, TNBC, and hormone receptor-positive (HR+) subtypes [31,32]. Recent developments in ADCs, nanotechnology-based drug delivery systems, and immunotherapy have contributed to precision oncology approaches, optimizing treatment efficacy while reducing systemic toxicity [33].

### 3.1. ADCs: Precision in Targeted Therapy

ADCs have emerged as a promising class of therapeutics, particularly for HER2-positive BC [34,35]. Trastuzumab emtansine (T-DM1) has been widely adopted, relying on lysosomal processing to release cytotoxic payloads [36]. Next-generation ADCs, such as trastuzumab duocarmazine (SYD985), incorporate protease-cleavable linkers and membrane-permeable drugs that enable a bystander effect, targeting antigen-negative neighboring cells [37].

Senescence-inducing therapies, including DNA-damaging agents and CDK4/6 inhibitors, can potentially enhance ADC efficacy by increasing lysosomal activity and cathepsin B-mediated linker cleavage [33]. Combination treatments with CDK4/6 inhibitors and SYD985 have demonstrated superior anti-tumor effects in patient-derived xenografts, suggesting a viable strategy for tumors with heterogeneous HER2 expression [38].

### 3.2. Nanotechnology in Drug Delivery

Conventional chemotherapy for metastatic BC is often limited by suboptimal drug bioavailability and systemic toxicity. Recent advancements in nanoparticle-based drug delivery have addressed these limitations. For instance, self-assembled cabazitaxel nanocrystals (PC/CNC) leverage protein corona-bridged natural targeting to enhance drug accumulation in primary tumors, circulating tumor cells, and metastatic lesions [39]. Similarly, mesoporous silica nanoparticle-based systems have been developed for pH-responsive targeted drug release, improving doxorubicin efficacy against BC cells [40].

Moreover, folic acid-functionalized starch-encapsulated copper oxide nanoparticles have shown promise in TNBC treatment by enhancing tumor penetration and inducing apoptosis via reactive oxygen species generation [41]. Another innovative approach involves chitosan/carbon quantum dots/Fe_2_O_3_ nanocomposites loaded with curcumin, designed for targeted drug release in tumor microenvironments [42].

Although nanotechnology and exosomal therapeutics hold tremendous promise in overcoming breast cancer resistance, their successful translation into widespread clinical use depends on resolving several formidable technical and practical obstacles [43].

Foremost among these is the issue of nanomaterial toxicity and biocompatibility. While nanoparticles are meticulously engineered for targeted drug delivery, their long-term safety profiles remain incompletely understood. Might these particles accumulate in non-target organs or provoke chronic inflammatory responses? Such possibilities underscore the urgent need for thorough investigations into their biodistribution, degradation, and clearance pathways to ensure safe in vivo application [44]. Achieving reliable elimination or biotransformation of these materials without harmful sequelae remains a significant scientific challenge.

Equally pressing is the matter of manufacturing scalability. Transforming these intricate nanoplatforms from laboratory-scale synthesis to large-scale, GMP-compliant production suitable for clinical trials—and eventually for routine clinical use—presents not only engineering complexities but also substantial economic hurdles [45]. Innovative approaches in process development and cost-effective manufacturing are therefore indispensable to bridge this gap between experimental promise and practical reality.

Targeting efficiency and specificity represent another persistent frontier. Despite advances in nanoparticle design, off-target accumulation continues to raise concerns about systemic toxicity. At the same time, insufficient tumor penetration can undermine therapeutic efficacy, emphasizing the importance of developing more sophisticated targeting ligands and responsive release systems tailored to the tumor microenvironment [46].

Turning to exosomal miRNA therapeutics, intriguing opportunities coexist with significant challenges. Exosomes offer a natural and versatile delivery system; however, their inherent instability in vivo poses a major barrier. Rapid clearance or degradation before reaching intended targets diminishes their therapeutic potential. This raises a crucial question: how might we enhance the circulation half-life and preserve the integrity of these valuable payloads? Strategies such as surface modification, protective encapsulation, or optimized storage protocols may offer viable solutions [47].

Ultimately, surmounting these scientific and logistical hurdles demands rigorous interdisciplinary collaboration and robust preclinical validation. Only through such concerted efforts can we hope to translate the remarkable potential of nanotechnology and exosome-based therapies into tangible improvements in breast cancer outcomes [48]. If these challenges can be addressed, the prospect of integrating these cutting-edge approaches into precision oncology could fundamentally transform patient care and survival.

### 3.3. Immunotherapy and TME Modulation

TNBCs frequently recur due to the development of chemoresistance. One promising approach involves targeting the immune checkpoint molecule CD47, which is overexpressed in TNBC cells following chemotherapy. Anti-CD47 monoclonal ADCs have enhanced cytotoxicity in TNBC models by facilitating macrophage-mediated phagocytosis and promoting natural killer cell activation [49]. Additionally, tumor metabolic profiling has been employed to identify immune-responsive BC subtypes, which exhibit enhanced sensitivity to immunotherapy and targeted agents such as sunitinib [50].

Neoadjuvant targeted therapy, which combines targeted agents with chemotherapy, has improved survival outcomes in HER2-positive BC. However, resistance remains challenging, with nearly 50% of patients failing to achieve a pathological complete response. In response, novel combination therapies have been developed, integrating photothermal therapy (PTT) and photodynamic therapy (PDT) with tyrosine kinase inhibitors such as pyrotinib. These strategies enhance oxidative stress-induced cell death, promoting ferroptosis and improving treatment efficacy [51]. Another multimodal approach exploits dual drug-loaded metal-phenolic networks for simultaneous chemodynamic therapy and magnetic resonance imaging (MRI)-guided treatment [52]. These nanoplatforms facilitate tumor-targeted drug release, amplify oxidative stress through glutathione depletion, and enhance chemotherapeutic efficacy via mitochondrial inhibition.

### 3.4. Addressing Drug Resistance in Targeted Therapy

Resistance to HER2-targeted therapy remains a significant barrier to sustained treatment efficacy. Recent real-world data suggest that primary resistance to anti-HER2 agents, including trastuzumab and T-DM1, correlates with poor prognosis [53]. TP53 mutations have been identified as potential resistance biomarkers, particularly in HER2-amplified and HER2-mutant non-amplified BC. Furthermore, molecular profiling studies indicate that TP53-mutant tumors exhibit differential responses to HER2 tyrosine kinase inhibitors, necessitating individualized treatment strategies [18].

The landscape of targeted BC therapy continues to evolve, with ADCs, nanoparticle-based delivery systems, immunotherapy, and multimodal treatment strategies demonstrating substantial therapeutic potential [54]. While these innovations offer new hope for improved outcomes, challenges such as drug resistance and tumor heterogeneity necessitate further research to optimize precision oncology approaches [55]. Looking ahead, future studies could fruitfully explore the integration of molecular profiling with advanced computational tools, such as artificial intelligence-driven predictive models, alongside personalized treatment regimens to maximize the clinical benefits of targeted BC therapies. A detailed analysis of AI methodologies and their specific implementation, however, is beyond the current scope and would be best addressed in a dedicated review.

Future research aims to optimize treatment efficacy and improve patient outcomes in BC therapy by leveraging combination strategies, integrating multimodal therapies, and applying personalized medicine approaches. Continued exploration of molecular mechanisms and therapeutic strategies will be essential in shaping the next generation of precision oncology.

Over recent years, the therapeutic landscape of breast cancer has been profoundly reshaped by advances in precision oncology, nanotechnology, and immune modulation. Yet, despite these significant strides, an essential question remains: how can the synergistic potential of these approaches be fully harnessed to deliver sustained clinical benefits [56].

Interestingly, emerging strategies such as multifunctional nanoplatforms and the innovative use of PROTACs targeting cancer stem cells embody this integrative vision. Unlike traditional monotherapies, these novel approaches are specifically designed to tackle two of the most formidable obstacles in oncology: tumor heterogeneity and the immunosuppressive TME. For example, nanoplatforms engineered to co-deliver chemotherapeutic agents alongside immune-stimulating molecules enable precise spatiotemporal control. This capability not only reduces systemic toxicity but also enhances localized immune activation—a crucial advantage, particularly when confronting ‘cold’ tumors that have historically resisted immunotherapeutic interventions [57].

However, translating these sophisticated technologies from laboratory innovation to clinical application is fraught with challenges. Success in this arena hinges on overcoming barriers such as scalable manufacturing processes, ensuring the long-term biocompatibility of nanomaterials, and optimizing targeting efficiency across diverse patient populations [58,59]. Likewise, although photoimmunotherapy shows significant promise in reprogramming the TME, its broader clinical adoption will depend on addressing technical limitations like restricted light penetration depth and the need for precise energy delivery to avoid collateral tissue damage [60].

This raises a compelling question: is it sufficient to focus solely on directly attacking tumor cells? Comparative analyses increasingly suggest otherwise. Therapeutic strategies aimed at modulating resistance pathways—either by disrupting aberrant signaling cascades or by re-educating immune cells—often demonstrate superior potential for achieving durable treatment responses compared to approaches targeting tumor cells alone [61]. Such findings underscore a growing realization in oncology that effective cancer therapy must address not just malignant cells but the complex ecosystem in which they exist.

**Figure 4:**
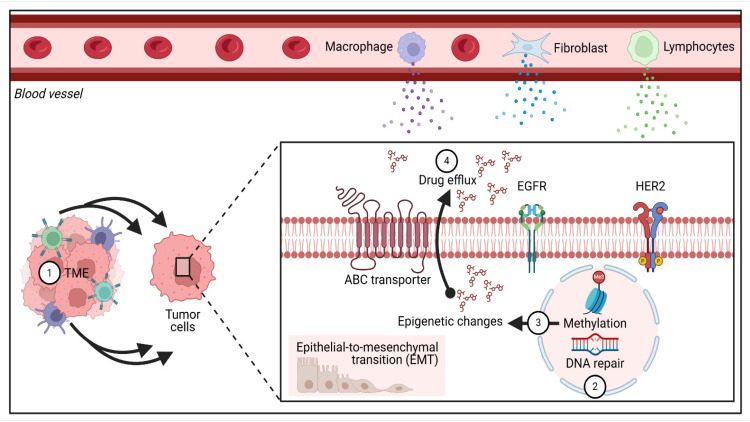
Mechanisms underlying drug resistance in breast cancer involve alterations in the tumor microenvironment, upregulated DNA repair processes, epigenetic changes, epithelial-to-mesenchymal transition (EMT), and elevated drug efflux activity.

Looking forward, future research efforts must adopt an interdisciplinary mindset. It is vital to not only refine these advanced therapeutic modalities but also to rigorously assess their cost-effectiveness and real-world applicability in varied healthcare systems [62]. After all, the ultimate measure of scientific progress lies in translating innovation into accessible and equitable patient care (Figure 4).

Integrating these emerging strategies into established treatment regimens holds great promise for transforming breast cancer therapy. By tackling longstanding challenges of efficacy and safety, such integration could herald a new era in patient-centered, precision oncology.

## 4. Overcome Drug Resistance in BC

Resistance mechanisms encompass genetic, epigenetic, and metabolic alterations that enable cancer cells to evade the cytotoxic effects of chemotherapy, targeted therapy, and immunotherapy [63]. Understanding these mechanisms is crucial for developing novel therapeutic strategies to counteract resistance and improve clinical outcomes.

### 4.1. Drug Resistance in BC

One of the primary contributors to chemotherapy resistance is the overexpression of ATP-binding cassette (ABC) transporters, such as P-glycoprotein (P-gp/ABCB1) and BC resistance protein (BCRP/ABCG2), which actively efflux chemotherapeutic agents, reducing intracellular drug accumulation and efficacy [64]. Studies have demonstrated that oxovanadium (IV) complexes can inhibit these transporters, restoring drug sensitivity in BC cells [65]. Similarly, 2,2-diphenethyl isothiocyanate (DPEITC) has been shown to suppress MDR1 expression and enhance topoisomerase inhibitor-induced cell death [66]. This restoration of drug sensitivity not only improves the therapeutic efficacy of existing chemotherapeutics but also opens new avenues for combination therapies that can effectively overcome resistance mechanisms in cancer treatment [67]. Research is ongoing to explore combinations of oxovanadium (IV) complexes with conventional chemotherapeutics, aiming to identify optimal dosing regimens and treatment schedules that maximize their synergistic effects while minimizing potential toxicity [68]. These investigations are crucial for developing personalized treatment strategies that can improve patient outcomes and reduce the likelihood of relapse in BC cases, ultimately contributing to more effective long-term management of the disease [69].

Metabolic reprogramming is increasingly recognized as a driver of drug resistance. Elevated cholesterol and mevalonate metabolism contribute to endocrine therapy resistance by activating the ERRα pathway, enhancing tumor growth and survival [70]. Moreover, cholesterol depletion through acetyl plumbagin has sensitized tamoxifen-resistant BC cells, suggesting that targeting lipid metabolism could be an effective approach [71]. Metabolic adaptations also enable cancer cells to resist oxidative stress and survive under hypoxic conditions, further contributing to drug resistance.

The TME is crucial in modulating drug response. Extracellular vesicles (EVs) have promoted resistance by transferring microRNAs that modulate key signaling pathways, such as Notch1/Hes1 [72]. Additionally, emerging drug delivery systems, such as nanoemulsions incorporating paclitaxel and erucin in frankincense oil, have shown promise in overcoming resistance through enhanced tumor penetration and sustained drug release [73]. These approaches aim to bypass the stromal barriers and to improve drug retention in tumor tissues.

Several intracellular signaling pathways contribute to resistance. The PI3K/AKT/mTOR axis is frequently dysregulated in estrogen receptor-positive (ER+) BC, leading to resistance against PI3K inhibitors. Network-based mathematical models have identified FOXO3 downregulation as a key resistance mechanism to alpelisib, a PI3Kα inhibitor, suggesting the potential for combination therapies with BH3 mimetics to enhance efficacy [74]. Similarly, the Wnt5a-mediated activation of cytochrome P450 (CYP) enzymes confers resistance to tamoxifen and paclitaxel in ER+ BC, independent of the PI3K pathway [75]. In TNBC, the mTOR inhibitor everolimus is rendered ineffective due to compensatory activation of the ERK pathway via SHOC2, highlighting the potential for combination therapy targeting both pathways [76]. Several mechanisms are proposed as follows.

#### 4.1.1. Tumor Necrosis Factor-Related Apoptosis-Inducing Ligand (TRAIL) Resistance

The resistance to TRAIL-induced apoptosis in BC is primarily driven by the downregulation of death receptors (DR4/DR5), overexpression of decoy receptors, and alterations in downstream apoptotic signaling components such as caspase-8 and FLIP [77]. Moreover, the immunosuppressive TME further dampens TRAIL responsiveness by promoting anti-apoptotic signaling and impairing immune effector cell function [78]. To address these challenges, novel nanoparticle-based systems have been engineered to modulate intracellular stress and reinstate TRAIL sensitivity.

One such promising strategy involves near-infrared (NIR) light-activated conjugated polymer nanoparticles (CPNs), which have been shown to induce endoplasmic reticulum (ER) stress and upregulate heat shock protein 70 (HSP70), thereby enhancing TRAIL gene expression and apoptotic signaling [79,80]. Importantly, these nanoparticles also co-deliver W-7, a calmodulin inhibitor, which potentiates caspase-8 activation—a critical initiator of the extrinsic apoptotic pathway. The dual-action design amplifies TRAIL signaling and circumvents intrinsic resistance mechanisms within the TME.

Such combinatorial nanoplatforms integrating photodynamic therapy and TRAIL potentiation exemplify a precision strategy to induce immunogenic apoptosis and sensitize resistant BC cells, particularly in triple-negative subtypes, where therapeutic options are limited.

#### 4.1.2. Role of Autophagy in Chemoresistance

Autophagy has emerged as a crucial factor in developing resistance to chemotherapeutic agents, particularly Taxol in TNBC [81]. Autophagy plays a dual role in cancer, acting as both a tumor suppressor and a mechanism of therapeutic resistance. In TNBC, elevated autophagic activity has been linked to resistance against chemotherapeutic agents like Taxol. The mammalian target of rapamycin (mTOR) pathway is central to autophagy regulation, with its inhibition leading to increased autophagic flux and enhanced cancer cell survival. MicroRNA-199a-3p (miR-199a-3p) has been identified as an upstream regulator suppressing mTOR signaling, promoting autophagy, and contributing to chemoresistance in TNBC cells. Formononetin (FMNT), a phytoestrogen derived from *Astragalus membranaceus,* has demonstrated potential in reversing Taxol resistance by targeting the miR-199a-3p/mTOR axis. By downregulating miR-199a-3p, FMNT restores mTOR activity, leading to a reduction in autophagic processes and sensitization of TNBC cells to Taxol. This suggests that modulation of autophagy through the miR-199a-3p/mTOR pathway could present a viable strategy to overcome chemoresistance in TNBC [82].

Furthermore, FMNT’s role extends beyond autophagy modulation. It has been shown to inhibit BC cell proliferation, invasion, and migration by suppressing signaling pathways such as NF-κB p65, p38, Akt, and p53. Additionally, FMNT influences the expression of long non-coding RNAs like AFAP1-AS1, which are implicated in cancer progression and drug resistance. These multifaceted actions underscore FMNT’s potential as a therapeutic agent in managing TNBC [83].

The interplay between miRNAs and autophagy is complex and significant in cancer therapy. MiR-199a-3p, in particular, has been recognized for its role in regulating autophagy-related genes, thereby affecting drug resistance in various cancers. Targeting such miRNAs offers a promising approach to modulate autophagy and enhance the efficacy of chemotherapeutic agents. In TNBC, where treatment options are limited, strategies that involve the inhibition of specific miRNAs to restore autophagic balance and counteract chemoresistance are of considerable interest. Further research into miRNA-mediated autophagy regulation could lead to the development of novel therapeutic interventions aimed at improving outcomes for patients with TNBC [84].

#### 4.1.3. Iron Modulation and Cancer Stem-like Phenotypes

Emerging research continues to elucidate the role of iron metabolism in promoting drug resistance in estrogen receptor-positive (ER+) BC. Specifically, mesenchymal stem cells (MSCs) have been shown to increase intracellular labile iron pools, promoting a shift towards cancer stem-like phenotypes that confer resistance to endocrine therapies such as tamoxifen and aromatase inhibitors [85]. Iron-mediated enhancement of the cancer stem cell (CSC) population facilitates survival under therapeutic stress, leading to recurrence and metastasis.

Mechanistically, iron accumulation activates key signaling pathways, including epithelial–mesenchymal transition (EMT), fostering a more invasive and therapy-resistant phenotype [86]. These findings underscore the significance of iron homeostasis in CSC biology and therapy resistance. Consequently, therapeutic interventions targeting iron metabolism have gained traction. For example, lysosomal iron chelators such as deferoxamine and novel nanoformulated agents have been reported to effectively deplete labile iron pools within CSCs, re-sensitizing them to endocrine therapy [87].

Furthermore, the interplay between iron metabolism and redox signaling represents a promising axis for therapeutic exploitation. Disrupting iron-mediated oxidative stress resilience in CSCs may suppress their proliferation and enhance the efficacy of ROS-generating chemotherapeutics. Overall, targeting iron modulation pathways holds substantial promise in reversing resistance and improving clinical outcomes in ER+ BC.

#### 4.1.4. Cancer Stem Cell (CSC)-Mediated Resistance

Recent research has elucidated the role of mesenchymal stem cells (MSCs) in promoting resistance to antiestrogen therapies in estrogen receptor-positive (ER+) BC. Direct interactions between MSCs and ER+ BC cells have been shown to induce cancer stem-like cell (CSC) phenotypes characterized by increased labile iron levels, which are associated with enhanced tumorigenicity and therapeutic resistance [88,89]. This iron accumulation contributes to activating EMT pathways, further reinforcing the CSC phenotype and complicating treatment outcomes [90,91].

Therapeutic strategies targeting iron metabolism have been explored to counteract this resistance mechanism. Iron chelators, such as deferoxamine (DFO), have demonstrated efficacy in depleting intracellular iron levels, thereby reducing CSC properties and restoring sensitivity to antiestrogen therapies [92,93]. Additionally, lysosomal iron-targeting compounds have shown promise in selectively inducing ferroptosis in CSCs, offering a novel approach to eliminating these resistant cell populations [89].

In TNBC, the CSC subpopulation significantly contributes to therapeutic resistance and metastasis. Recent studies have focused on developing conjugates that combine lapatinib derivatives with CSC inhibitors. These conjugates have effectively reversed resistance by concurrently inhibiting the AKT/ERK and Wnt/β-catenin signaling pathways, which are crucial for CSC maintenance and survival. This dual-targeting strategy underscores the potential of combination therapies in overcoming CSC-mediated resistance in aggressive BC subtypes [94].

Furthermore, the inhibition of lysosomal channels, such as TRPML1, has been identified as a potential therapeutic target. Salinomycin, an antibiotic with anticancer properties, has been shown to modulate lysosomal iron homeostasis by acting on TRPML1 channels, leading to the elimination of BC cells and CSCs [95]. This approach highlights the significance of targeting lysosomal function in addressing iron metabolism-related drug resistance.

Collectively, these findings emphasize the importance of targeting iron metabolism and associated signaling pathways in overcoming CSC-mediated drug resistance in BC. Meanwhile, integrating iron chelators, lysosomal iron-targeting agents, and pathway-specific inhibitors into therapeutic regimens holds promise for enhancing treatment efficacy and reducing relapse rates in resistant BC cases.

#### 4.1.5. Wnt/β-Catenin Signaling and OTULIN Phosphorylation

In recent years, mounting evidence has confirmed the Wnt/β-catenin signaling pathway as a central mediator of BC chemoresistance, particularly in triple-negative and stem cell-like phenotypes. OTULIN, a deubiquitinase, has been identified as a key regulator of β-catenin stability through its interaction with ABL1, which phosphorylates OTULIN under genotoxic stress, preventing β-catenin degradation and promoting resistance [96].

Chen, Gao [97] demonstrated that pharmacological inhibition of OTULIN sensitized resistant BC cells to doxorubicin by destabilizing β-catenin and reducing downstream survival signaling. Targeting β-catenin directly has also shown therapeutic potential; Zhou, Liu [98] reported that silencing β-catenin restored tamoxifen sensitivity in resistant models. Notably, Park, Lee [99] underscored that Wnt/β-catenin activation enhances stemness and epithelial–mesenchymal transition, further promoting therapy resistance.

Moreover, Nguyen, Tran [100] showed that β-catenin inhibition reduced cancer stemness markers and sensitized tumors to chemotherapy in patient-derived xenografts. These findings collectively support the therapeutic rationale for disrupting OTULIN-mediated β-catenin activation as a strategy to overcome resistance in BC.

#### 4.1.6. Fibroblast-Mediated Resistance in HER2+ BC

Fibroblasts within the TME significantly contribute to drug resistance by secreting factors that modify signaling pathways in neighboring cancer cells. In HER2-positive BC, fibroblast-secreted proteins such as PAI-1 and PLK1 have been shown to induce resistance to HER2-targeted therapies, including lapatinib [101]. Mechanistically, PAI-1 enhances extracellular matrix remodeling and promotes epithelial–mesenchymal transition (EMT), facilitating drug resistance and metastatic progression [102]. Concurrently, PLK1 activation supports cancer cell proliferation and survival under therapeutic pressure by driving mitotic checkpoint adaptation [103]. Blockade of PLK1 or PAI-1 in combination with HER2 inhibitors has restored sensitivity in preclinical models, suggesting a synergistic therapeutic strategy [104].

Further investigations have demonstrated that fibroblasts can modulate tumor immune evasion and stromal stiffness, creating a physical and biochemical barrier to drug penetration. Targeting these microenvironmental elements has emerged as a viable approach, with dual PLK1/HER2 inhibition showing efficacy in overcoming stromal-induced resistance in vivo [105]. These findings collectively emphasize the need for a paradigm shift towards microenvironment-focused therapies in HER2+ BC.

#### 4.1.7. Single-Cell Transcriptomics and Drug Resistance Acquisition

Recent advancements in single-cell RNA sequencing (scRNA-seq) have significantly enhanced our understanding of the molecular mechanisms underlying drug resistance in BC. By enabling gene expression analysis at the individual cell level, scRNA-seq has revealed the heterogeneity and dynamic transitions that contribute to resistance development [106,107].

In a study by Iida and Okada (2024) [108], time-series scRNA-seq data from tamoxifen-treated MCF-7 cells were analyzed using pseudo time trajectory analysis. This approach identified five distinct subpopulations encompassing tamoxifen-sensitive and -resistant cells. Notably, the study highlighted the role of RPS6KB1, a gene associated with poor prognosis in estrogen receptor-positive BC, exhibiting multistable expression states during the acquisition of drug resistance. Bifurcation analysis further elucidated the regulatory mechanisms of such key genes, providing insights into potential therapeutic targets.

Integrating scRNA-seq with single-cell ATAC sequencing (scATAC-seq) has also shed light on the epigenetic factors contributing to tamoxifen resistance. Fang et al. [109] conducted an integrated analysis that uncovered distinct chromatin accessibility patterns and transcription factor activities associated with resistant cell populations. This comprehensive approach offers a deeper understanding of tumor heterogeneity and identifies novel biomarkers for predicting therapeutic outcomes.

Furthermore, scRNA-seq has been instrumental in identifying molecular biomarkers predictive of late progression in patients undergoing CDK4/6 inhibitor therapy. Luo et al. [110] analyzed metastatic tumors from hormone receptor-positive, HER2-negative BC patients and identified specific gene expression profiles correlating with progression timelines. These findings underscore the potential of scRNA-seq in developing personalized treatment strategies.

Taken together, these studies demonstrate the power of single-cell transcriptomics in elucidating the complex mechanisms of drug resistance acquisition. The insights gained pave the way for developing targeted therapies and personalized medicine approaches aimed at overcoming resistance to BC treatment.

### 4.2. Strategies to Overcome Drug Resistance

Several studies have explored combinational approaches to counteract resistance. For instance, the combination of apatinib and vinorelbine has shown efficacy in refractory HER2-negative BC, with metabolomic analyses identifying disrupted metabolic pathways contributing to resistance [111]. Similarly, a ketogenic diet combined with melatonin has demonstrated the ability to reverse resistance to cisplatin and vincristine through apoptosis induction and angiogenesis inhibition [112]. Such approaches highlight the importance of simultaneously targeting multiple resistance pathways.

Cancer stem cells (CSCs) contribute to long-term tumor persistence and drug resistance. GALNT14, in association with GDF-15, promotes stemness and resistance via the β-catenin signaling pathway [113]. Moreover, non-genetic heterogeneity and phenotypic plasticity allow reversible resistance states, emphasizing the importance of targeting EMT in treatment-resistant BC [114]. Inhibiting EMT-associated transcription factors, such as ZEB1 and Snail, has been proposed as a potential therapeutic strategy.

Advancements in nanotechnology have led to the development of drug-delivery systems designed to overcome resistance. Matrix metalloproteinase-2-responsive surface-changeable liposomes have been shown to enhance drug penetration in TNBC, improving paclitaxel and tariquidar efficacy [115]. Additionally, 3D bioprinting models of drug-resistant BC spheroids have provided a robust platform for screening novel therapeutic agents [116,117]. These technological innovations facilitate precise drug targeting and reduce systemic toxicity.

#### 4.2.1. Nanotechnology-Based Drug Delivery

Nanoformulations are emerging as effective strategies to bypass efflux pumps and improve drug accumulation in resistant cells. A two-step nano-celastrol formulation successfully reversed MDR in BC models by inhibiting drug efflux transporters and inducing apoptosis through ERK/JNK signaling [118]. Additionally, pH-sensitive nanoparticles have been designed to co-deliver chemotherapeutic agents with resistance modulators, thereby enhancing drug efficacy [119].

#### 4.2.2. Targeting Cancer Stem Cells (CSCs)

BC stem cells (BCSCs) are crucial in therapy resistance and tumor relapse. Strategies targeting CSCs include the inhibition of stemness-related pathways, such as GALNT14/GDF-15-mediated β-catenin signaling [120] and the use of adenovirus-mediated gene therapy to restore tumor-suppressive functions [121]. Notably, a study demonstrated that restoring microRNA-34a expression suppresses the ABCC1 gene, effectively overcoming resistance in human BC cell lines [122].

#### 4.2.3. Targeting Apoptotic and Survival Pathways

Evasion of apoptosis is a hallmark of drug-resistant BC. Nuclear XIAP has been linked to chemoresistance through NFκB activation and ubiquitination processes, highlighting its potential as a therapeutic target [123]. Similarly, a ketogenic diet combined with melatonin was shown to reverse resistance to cisplatin and vincristine by promoting apoptosis and reducing angiogenesis [124].

#### 4.2.4. Overcoming Metabolic Adaptations

Metabolic plasticity allows BC cells to evade therapy by reprogramming energy pathways. Cholesterol and mevalonate metabolism were crucial in sustaining drug resistance by activating the ERRα pathway [125]. Inhibiting these metabolic pathways presents a promising strategy to enhance chemotherapy sensitivity.

Integrating genomic and metabolomic profiling has become pivotal in developing personalized therapeutic strategies for BC. By identifying specific resistance mechanisms at the individual patient level, clinicians can tailor interventions to enhance treatment efficacy. Artificial intelligence (AI)-driven predictive models are increasingly utilized to stratify patients based on molecular signatures, optimizing drug regimens accordingly. For instance, AI algorithms have been employed to analyze histopathological images, facilitating the detection of genomic and proteomic biomarkers directly from routine hematoxylin and eosin-stained slides, thereby supporting treatment decisions without costly molecular assays [126].

Patient-derived organoid (PDO) models are emerging as powerful tools for preclinical testing of targeted therapies. These three-dimensional cultures faithfully recapitulate the heterogeneity and architecture of the original tumors, allowing for the evaluation of drug responses in a patient-specific context. Recent studies have demonstrated the utility of PDOs in modeling the TME and predicting individualized drug responses, thereby guiding personalized treatment strategies [127].

In addressing drug resistance, combination therapies that include metabolic pathway inhibitors have shown promise. For example, the use of PI3K inhibitors in combination with endocrine therapy has been explored to overcome resistance in hormone receptor-positive BC [128]. Additionally, targeted delivery systems utilizing nanotechnology are being developed to enhance the precision and efficacy of chemotherapeutic agents, minimizing off-target effects and improving patient outcomes [129].

CSC-targeting strategies are also being investigated to mitigate resistance mechanisms. Emerging agents that inhibit key CSC signaling pathways, such as Wnt, Notch, and Hedgehog, are under preclinical and clinical evaluation, offering potential avenues to eradicate CSCs and prevent tumor relapse [130].

Future research should focus on integrating multi-omics data to develop precision medicine approaches tailored to individual resistance profiles. The convergence of genomic, transcriptomic, and metabolomic analyses, coupled with advanced computational models, holds the promise of identifying novel therapeutic targets and optimizing treatment strategies for BC patients.

## 5. Therapeutic Strategies

The growing understanding of molecular resistance mechanisms has spurred research into targeted interventions that may improve clinical outcomes [131]. Several pathways, including epigenetic modifications, metabolic reprogramming, and TME interactions, contribute to drug resistance, necessitating further multifaceted therapeutic strategies.

### 5.1. Targeted Approaches for Overcoming Drug Resistance

One critical factor in resistance to HER2-targeted therapy is YES1 amplification, which plays a key role in trastuzumab and trastuzumab-emtansine (T-DM1) resistance. Persistent YES1 activation promotes tumor growth despite HER2 inhibition, and targeting YES1 with Src inhibitors such as dasatinib has restored T-DM1 sensitivity in preclinical models, suggesting a potential therapeutic avenue [132]. Additionally, HER2-positive brain metastases remain a significant clinical hurdle due to poor blood–brain barrier permeability. Neuregulin-1 (NRG1) has been shown to drive resistance by activating ErbB3/PI3K-AKT signaling, counteracting the effects of HER2 inhibitors. Poziotinib, a brain-penetrable receptor tyrosine kinase inhibitor, has demonstrated efficacy in overcoming HER2-positive brain metastases, marking a significant advancement [133].

In estrogen receptor-positive (ER+) BC, endocrine resistance is a significant challenge, often driven by heightened ER transcriptional activation. High ERα activity paradoxically enables tumor cells to survive estrogen deprivation while simultaneously sensitizing them to estradiol-induced apoptosis. Cycling between estrogen deprivation and 17β-estradiol treatment has been proposed as a novel endocrine therapy strategy to sustain long-term tumor suppression [134]. Additionally, activation of the RON/PI3K pathway has been implicated in ESR1-mutant tumors, which commonly emerge following aromatase inhibitor therapy. Inhibiting RON/PI3K pathway, either alone or in combination with endocrine therapy, has shown promise in suppressing ESR1-mutant metastatic BC and overcoming palbociclib resistance [135].

Resistance to CDK4/6 inhibitors has emerged as another critical issue. The upregulation of PEG10 enhances EMT and suppresses key regulators of the cell cycle, promoting resistance. Targeting PEG10 with antisense oligonucleotides or siRNA in combination with palbociclib has shown promise in overcoming this resistance [131]. Additionally, PKMYT1, a kinase associated with the G2/M checkpoint, has been identified as a biomarker of CDK4/6 inhibitor resistance. The PKMYT1 inhibitor lunresertib, particularly in combination with gemcitabine, has shown potential in selectively targeting CDK4/6-resistant tumors [136].

TNBC is mostly aggressive and challenging due to its lack of targeted therapies and frequent resistance to chemotherapy. Resistance in TNBC is often mediated by SRC-family kinases (SFKs) and MAPK/ERK pathway activation, with patient-derived xenograft models indicating that SFK inhibitors, particularly those targeting SRC, FYN, and YES1, may help resensitize resistant tumors [137]. Furthermore, the IκB kinase (IKK) pathway has been implicated in resistance to epidermal growth factor receptor (EGFR) inhibitors, with IKK inhibition enhancing the efficacy of gefitinib [138]. In addition, exosome-mediated communication has been shown to facilitate therapeutic resistance in TNBC. Exosomes secreted by radioresistant BC cells transfer resistance traits to naïve recipient cells, underscoring the potential of targeting exosome pathways to mitigate resistance [139].

### 5.2. Emerging Therapeutic Strategies

Metabolic reprogramming has also been implicated in therapy resistance across BC subtypes. The RNA-binding protein RPS15 enhances homologous recombination repair, allowing cancer cells to survive genotoxic therapies such as PARP inhibitors and radiation [140]. Additionally, FOXF2 has been identified as a key factor in endocrine resistance, orchestrating chromatin remodeling to activate survival pathways. Targeting FOXF2 and its coactivator BRD4 could enhance sensitivity to endocrine therapy [141]. Another metabolic regulator, acyl-CoA synthetase 4 (ACSL4), is involved in lipid metabolism and steroidogenesis, with pharmacological inhibition reducing tumor growth and improving chemotherapeutic efficacy [142].

Tamoxifen resistance remains a significant issue in ER+ BC, with both genomic and epigenetic alterations playing crucial roles. Genome-wide analyses identified five biological pathways predicting tamoxifen failure, facilitating patient stratification for alternative therapies [143]. Additionally, miR-330-3p has been shown to drive tamoxifen resistance by suppressing histone deacetylase 4 (HDAC4), and targeting miR-330-3p has been proposed as a strategy to restore tamoxifen sensitivity [144]. Similarly, the hormone-regulated protein semaphorin 7A (SEMA7A) has been implicated in driving endocrine therapy resistance by promoting survival signaling and metastatic potential, suggesting that targeting this pathway could improve treatment outcomes [145].

The development of novel combination therapies has shown promise in reversing resistance. The kinome-wide RNAi screen identified WEE1 as a key mediator of ABT-199 resistance in BC, with WEE1 inhibition significantly enhancing sensitivity to BCL-2 inhibitors [146]. Additionally, PLK1 inhibition has been found to reverse resistance to BET inhibitors in TNBC, representing a potential therapeutic approach for BETi-resistant tumors [147]. Another novel approach involves Trop-2-targeting ADCs, such as sacituzumab govitecan, which have demonstrated clinical efficacy. Interestingly, tamoxifen treatment has increased Trop-2 expression, potentially enhancing ADC efficacy in luminal BC [148].

Multidrug resistance remains a persistent challenge across BC subtypes, often mediated by efflux transporters such as MDR1 and MRP1. Novel selenium-based compounds, such as selenoesters EDAG-1 and EDAG-8, have been found to inhibit these transporters, increasing drug retention within tumor cells and enhancing chemotherapy efficacy [149]. Additionally, the chromatin-modifying protein CHMP4C has been implicated in doxorubicin resistance, with its suppression enhancing drug sensitivity and reducing tumor growth, highlighting chromatin modification as a promising therapeutic target [150].

The growing understanding of the molecular mechanisms underlying drug resistance in BC underscores the importance of precision medicine approaches. Future research should focus on integrating genetic, epigenetic, and metabolic profiling to develop personalized therapeutic strategies. By targeting key regulators of therapy resistance, such as RAC1, SRC-family kinases, IGF1R, YES1, and FOXF2, in combination with metabolic and immune-based interventions, novel treatment paradigms can be established to improve patient outcomes in BC.

#### 5.2.1. Nanotechnology-Based Drug Delivery

Nanotechnology-based drug delivery has emerged as a revolutionary strategy for overcoming drug resistance, particularly in cancer therapy. Nanoparticles offer enhanced drug stability, targeted delivery, and increased intracellular uptake, crucial for improving treatment efficacy. One of the critical limitations of conventional chemotherapy is non-specific distribution, leading to systemic toxicity and suboptimal therapeutic outcomes. Nanoparticles facilitate controlled drug release and specific tumor targeting via active and passive targeting mechanisms, such as the enhanced permeability and retention (EPR) effect [151].

Polymeric nanoparticles, lipid-based carriers, and inorganic nanocarriers are among the most explored nanoplatforms for drug delivery. Polymeric nanoparticles, such as poly-lactic-co-glycolic acid (PLGA) and polyethylene glycol (PEG)-conjugated carriers, have been extensively studied due to their biocompatibility and controlled release properties [152]. Lipid nanoparticles, particularly liposomes and solid lipid nanoparticles (SLNs), have demonstrated significant potential in enhancing drug bioavailability and penetration across biological barriers [153]. Additionally, inorganic nanoparticles, such as gold and mesoporous silica nanoparticles, have been employed to enhance drug delivery through photothermal effects and improved endocytosis [154].

A recent study developed poly-l-lactic-acid (PLLA) porous scaffolds as 3D BC models, demonstrating increased resistance in three-dimensional cultures compared to traditional two-dimensional models [155]. This underscores the importance of biomimetic models in drug screening and therapeutic optimization. Furthermore, surface modifications such as targeting ligands, aptamers, and antibodies significantly improve the specificity of nanocarriers. For example, folate-functionalized nanoparticles have enhanced uptake in folate receptor-overexpressing tumors, improving therapeutic index and reducing off-target effects [156].

However, despite the promising potential of nanotechnology in drug delivery, several challenges remain, including potential toxicity, regulatory hurdles, and large-scale manufacturing issues (reviewed in [157]). The long-term safety of nanoparticles remains a concern, as their accumulation in non-target organs may lead to unforeseen side effects. Moreover, translating preclinical findings into clinical applications requires rigorous evaluation through standardized protocols and regulatory approval processes [158,159]. Addressing these challenges will be crucial in maximizing the therapeutic potential of nanotechnology-based drug delivery systems.

#### 5.2.2. Photoimmunotherapy and Macrophage Reprogramming

Photoimmunotherapy (PIT) has emerged as a novel therapeutic approach to combat tumor resistance by integrating phototherapy with immune modulation. This approach leverages near-infrared (NIR) light-activated nanoparticles conjugated with monoclonal antibodies to induce selective cytotoxicity while triggering an immune response [160]. The highly selective NIR-PIT minimizes damage to normal tissues while enhancing antigen presentation and immune activation.

Recent studies have demonstrated the efficacy of PIT in aggressive BCs, particularly in TNBC, which is notorious for its resistance to conventional therapies. For instance, cetuximab-conjugated gold nanorods combined with NIR irradiation effectively reduced TNBC resistance by reprogramming tumor-associated macrophages [161]. Tumor-associated macrophages (TAMs) play a dual role in the tumor microenvironment, often supporting tumor growth and immune evasion. PIT enhances immune-mediated tumor eradication by shifting macrophages from the pro-tumor M2 phenotype to the anti-tumor M1 phenotype [162].

Moreover, PIT has been shown to disrupt the tumor extracellular matrix, improving drug penetration and reducing hypoxia-mediated resistance. Due to their photothermal and photodynamic properties, gold-based nanoparticles have demonstrated superior efficacy in PIT applications, leading to enhanced immune priming and T-cell infiltration [151]. Another critical advantage of PIT is its ability to generate immunogenic cell death (ICD), which releases tumor-associated antigens that further stimulate an adaptive immune response [163,164].

Despite these advancements, several limitations need to be addressed for clinical translation. One of the primary challenges is the TME heterogeneity, which may influence treatment response variability [165]. Optimizing light penetration in deep-seated tumors remains a technical challenge, necessitating the development of advanced NIR-responsive nanoplatforms [166]. Overcoming these barriers through advanced material engineering and combination therapies could significantly enhance the efficacy and applicability of PIT.

#### 5.2.3. Targeting Exosomal miRNAs

Exosomal miRNAs have gained attention as critical mediators of drug resistance in cancer. Exosomes, small extracellular vesicles, facilitate intercellular communication by transferring bioactive molecules, including miRNAs, that regulate gene expression and cellular functions [167,168]. These vesicles contribute to tumor progression by promoting EMT, modulating immune responses, and enhancing drug efflux mechanisms.

A recent study identified miR-9-5p as a key factor in tamoxifen resistance, as it regulates drug efflux transporters and apoptotic pathways [169]. This finding highlights the potential of targeting exosomal miRNA pathways to overcome resistance in hormone receptor-positive BCs. Other studies have identified additional miRNAs, such as miR-21 and miR-222, which are implicated in chemoresistance by modulating apoptosis and cell survival signaling pathways [170].

Blocking exosomal miRNA signaling represents a promising therapeutic strategy. One approach involves using synthetic anti-miRNAs (antagomiRs) to neutralize oncogenic miRNAs [171]. Additionally, exosome inhibitors, such as GW4869, have been investigated to disrupt exosomal biogenesis and secretion, thereby reducing the transfer of resistance-related miRNAs [172]. Furthermore, engineered exosomes loaded with tumor-suppressive miRNAs have been explored to counteract drug resistance [173].

However, the translation of exosome-targeted therapies faces several hurdles, including the complexity of exosome isolation, potential off-target effects, and stability of miRNA-based drugs [174]. Standardization of exosome isolation methods and improving delivery platforms are critical for the clinical success of miRNA-targeting approaches. Future research should focus on optimizing exosomal engineering techniques and validating the efficacy of exosome-based interventions in clinical settings. Table 1 summarizes the therapeutic approaches, mechanisms, and outcomes for BC treatment.

## 6. Conclusions

Overcoming drug resistance in BC is paramount to achieving durable clinical responses and improving patient survival, especially in refractory subtypes such as TNBC. The convergence of nanotechnology, precision oncology, and immune modulation provides a transformative framework to target resistance at multiple biological levels. Researchers are reshaping the therapeutic landscape by engineering multifunctional nanoplatforms capable of reprogramming tumor metabolism, enhancing immune responses, and delivering therapeutic agents with precision. Advances in photoimmunotherapy, biomarker-driven targeting, and exosomal miRNA inhibition represent promising frontiers. These innovations deepen our understanding of resistance mechanisms and open new avenues for clinical translation and individualized treatment regimens. As the field moves forward, the integration of computational modeling, omics technologies, and patient-derived models will be essential to refining these approaches. We recommend prioritizing translational research efforts that combine nanotechnology and immunotherapy for personalized BC resistance management.

## Figures and Tables

**Table 1:** Summary of Therapeutic Approaches, Mechanisms, and Outcomes for Breast Cancer Treatment.

Therapeutic Agent/Approach	Mechanism/Regimen Type	Primary Outcome/Effect
**Ultrasound-responsive nanobubbles co-loaded with chlorin-e6 and paclitaxel**	Chemotherapy, sonodynamic therapy, and immune activation via cGAS-STING pathway	Suppressing tumor progression and enhancing CD8^+^ T cell infiltration in TNBC models
**Single-atom iron nanozymes (Fe-N-C SAzymes)**	Mimicking peroxidase activity to remodel tumor metabolism and immune landscape	Potentiating chemodynamic therapy (CDT) and photothermal therapy (PTT), eliminating immunosuppressive myeloid-derived suppressor cells
**Gold nanorods and CXCR4 antagonist peptide E5 (AuNRs-E5)**	Disrupting tumor proliferation, initiating endoplasmic reticulum stress, promoting dendritic cell maturation	Long-term immune memory and suppression of TNBC recurrence
**IC/IR820 nanoparticles (combination of chemotherapy with photothermal and immune stimulation)**	Inducing robust autophagy-dependent immunogenic cell death	Inducing robust autophagy-dependent immunogenic cell death
**Nano–Micro-Sera-based fibrin implant (photothermal agents, chemotherapeutics, and immune agonists)**	Multi-agent, locally administered system to prevent TNBC relapse post-surgery	Enhanced immune infiltration at the tumor site, prevented local recurrence, and achieved remarkable tumor suppression
**Pt(IV)/CQ/PFH constructs (nanoparticles)**	Inhibiting protective autophagy while reprogramming innate immune metabolism	Boosting maturation of dendritic cells and polarization of macrophages to pro-inflammatory phenotypes
**Multi-modal nanoplatforms (immunogenic cell death inducers, e.g., mitoxantrone, immune adjuvants, e.g., TLR7/8 agonists, and photothermal agents)**	Repolarizing tumor-associated macrophages, enhancing dendritic cell activation, converting cold tumors into immunologically hot ones	Enhanced clinical efficacy in BC, particularly TNBC
**CDK4/6 inhibitors (palbociclib, abemaciclib)**	Molecularly targeted therapy for HR+/HER2− metastatic BC	Median progression-free survival (PFS) of 5.3 months in heavily pretreated patients
**mTOR inhibitors (everolimus)**	Molecularly targeted therapy for HR+/HER2− metastatic BC	Median progression-free survival (PFS) of 5.3 months in heavily pretreated patients
**Selective Estrogen Receptor Degraders (SERDs)**	Addressing ESR1 mutations in endocrine therapy resistance	More effective inhibition of ESR1 mutations
**Mitochondria-targeted organic nanoparticles**	Mild photothermal therapy	Improved cancer cell apoptosis, reduced tumor metastasis
**Dual-targeted cationic microbubbles**	Enhanced gene therapy delivery	Improved tumor suppression
**Aptamer-PROTAC approach targeting ERα**	Degrading mutant ERα variants that drive resistance to tamoxifen	Overcoming endocrine resistance in ERα-positive BC
**Trastuzumab emtansine (T-DM1)**	ADC: Lysosomal processing to release cytotoxic payloads	Widely adopted for HER2-positive BC
**Trastuzumab duocarmazine (SYD985)**	Next-generation ADC: Incorporates protease-cleavable linkers and membrane-permeable drugs, enabling a bystander effect	Targeting antigen-negative neighboring cells
**Self-assembled cabazitaxel nanocrystals (PC/CNC)**	Protein corona-bridged natural targeting	Enhancing drug accumulation in primary tumors, circulating tumor cells, and metastatic lesions
**Mesoporous silica nanoparticle-based systems**	pH-responsive targeted drug release	Improving doxorubicin efficacy against BC cells
**Folic acid-functionalized starch-encapsulated copper oxide nanoparticles**	Enhancing tumor penetration and inducing apoptosis via reactive oxygen species generation	Promising in TNBC treatment
**Chitosan/carbon quantum dots/Fe_2_O_3_ nanocomposites loaded with curcumin**	Targeted drug release in tumor microenvironments	Targeted drug release in tumor microenvironments
**Anti-CD47 monoclonal ADCs**	Targeting immune checkpoint molecule CD47; facilitating macrophage-mediated phagocytosis and promoting natural killer cell activation	Enhanced cytotoxicity in TNBC models
**Photothermal therapy (PTT) and Photodynamic therapy (PDT) with tyrosine kinase inhibitors (e.g., pyrotinib)**	Enhancing oxidative stress-induced cell death	Promoting ferroptosis and improving treatment efficacy in HER2-positive BC
**Dual drug-loaded metal–phenolic networks**	Simultaneous chemodynamic therapy and MRI-guided treatment; amplifying oxidative stress through glutathione depletion; enhancing chemotherapeutic efficacy via mitochondrial inhibition	Facilitate tumor-targeted drug release, amplify oxidative stress, and enhance chemotherapeutic efficacy
**Oxovanadium (IV) complexes**	Inhibiting ABC transporters (P-gp/ABCB1 and BCRP/ABCG2)	Restoring drug sensitivity in BC cells, improving therapeutic efficacy of existing chemotherapeutics
**2,2-diphenethyl isothiocyanate (DPEITC)**	Suppressing MDR1 expression	Enhancing topoisomerase inhibitor-induced cell death
**Acetyl plumbagin**	Cholesterol depletion	Sensitized tamoxifen-resistant BC cells
**Nanoemulsions incorporating paclitaxel and erucin in frankincense oil**	Enhanced tumor penetration and sustained drug release	Overcoming resistance
**Combination therapies with BH3 mimetics (for PI3Kα inhibitor resistance)**	Targeting FOXO3 downregulation	Enhancing efficacy against resistance to PI3K inhibitors
**Near-infrared (NIR) light-activated conjugated polymer nanoparticles (CPNs) with W-7**	Inducing endoplasmic reticulum (ER) stress, upregulating heat shock protein 70 (HSP70), enhancing TRAIL gene expression and apoptotic signaling, potentiating caspase-8 activation	Enhancing TRAIL sensitivity and inducing immunogenic apoptosis in resistant BC cells, particularly in TNBC
**Formononetin (FMNT)**	Downregulating miR-199a-3p, restoring mTOR activity, inhibiting BC cell proliferation, invasion, and migration	Reduction in autophagic processes, sensitization of TNBC cells to Taxol, potential for reversing Taxol resistance
**Lysosomal iron chelators (e.g., deferoxamine) and novel nanoformulated agents**	Depleting labile iron pools within CSCs	Re-sensitizing CSCs to endocrine therapy, suppressing CSC proliferation, enhancing efficacy of ROS-generating chemotherapeutics
**Lapatinib derivatives with CSC inhibitors (conjugates)**	Concurrently inhibiting AKT/ERK and Wnt/β-catenin signaling pathways	Reversing resistance in TNBC by overcoming CSC-mediated resistance

## Data Availability

All data generated are presented in the current MS.

## Abbreviations

ABC: ATP-binding cassette; ABCG2: ATP-binding cassette sub-family G member 2; ABCC1: ATP-binding cassette sub-family C member 1; ABL1: abelson tyrosine kinase 1; ADC: antibody–drug conjugate; AFAP1-AS1: actin filament associated protein 1 antisense RNA 1; AI: artificial intelligence; AKT: protein kinase B; ATAC-seq: assay for transposase-accessible chromatin using sequencing; BC: breast cancer; BCSC: BC stem cell; BETi: bromodomain and extra-terminal inhibitor; BRD4: bromodomain-containing protein 4; CD47: cluster of differentiation 47; CDK4/6: cyclin-dependent kinase 4/6; CSC: cancer stem cell; CPNs: conjugated polymer nanoparticles; CXCR4: C-X-C chemokine receptor type 4; DPEITC: 2,2-diphenethyl isothiocyanate; DFO: deferoxamine; DNA: deoxyribonucleic acid; EGFR: epidermal growth factor receptor; EMT: epithelial–mesenchymal transition; ER+: estrogen receptor positive; ERRα: estrogen-related receptor alpha; ERK: extracellular signal-regulated kinase; ESR1: estrogen receptor 1; EPR: enhanced permeability and retention; EVs: extracellular vesicles; FDA: food and drug administration; FMNT: formononetin; FOXF2: forkhead box F2; GDF-15: growth differentiation factor 15; GSTP1: glutathione S-transferase P1; HER2: human epidermal growth factor receptor 2; HSP70: heat shock protein 70; ICD: immunogenic cell death; IL-6: interleukin-6; IMQ: imiquimod; mTOR: mammalian target of rapamycin; MDSC: myeloid-derived suppressor cell; miRNA: microRNA; MRI: magnetic resonance imaging; MTO: mitoxantrone; MSC: mesenchymal stem cell; NIR: near-infrared; NGS: next-generation sequencing; OTULIN: OTU deubiquitinase with linear linkage specificity; PAI1: plasminogen activator inhibitor 1; PDO: patient-derived organoid; PFH: perfluorohexane; PI3K: phosphoinositide 3-kinase; PIT: Photoimmunotherapy; PLGA: poly(lactic-co-glycolic acid); PLK1: polo-like kinase 1; PMCID: PubMed central identifier; PNS: plasmonic nanosera; PROTACs: proteolysis-targeting chimeras; PTT: photothermal therapy; PDT: photodynamic therapy; RPS6KB1: ribosomal protein S6 kinase B1; ROS: reactive oxygen species; SAzyme: single-atom nanozyme; scATAC-seq: single-cell assay for transposase-accessible chromatin using sequencing; scRNA-seq: single-cell RNA sequencing; SEMA7A: Semaphorin 7A; SERDs: selective estrogen receptor degraders; SFKs: Src family kinases; SLNs: solid lipid nanoparticles; SYD985: trastuzumab duocarmazine; T-DM1: trastuzumab emtansine; TAM: tumor-associated macrophage; TCGA: the cancer genome atlas; TILs: tumor-infiltrating lymphocytes; TLR: toll-like receptor; TNBC: triple-negative breast cancer; TRAIL: tumor necrosis factor-related apoptosis-inducing ligand; TME: tumor microenvironment; TP53: tumor protein p53; TRPML1: transient receptor potential mucolipin 1; Wnt: wingless/integrated pathway; YES1: YES proto-oncogene 1.
